# Comparing the phonological, musical, and general cognitive profiles of early-emerging poor, average, and good readers of Chinese

**DOI:** 10.3389/fpsyg.2025.1563491

**Published:** 2025-08-29

**Authors:** William Choi, Alfredo Bautista, Siu-Hang Kong, Veronica Ka Wai Lai

**Affiliations:** ^1^Academic Unit of Human Communication, Learning, and Development, The University of Hong Kong, Hong Kong, China; ^2^Speech and Music Perception Laboratory, The University of Hong Kong, Hong Kong, China; ^3^Centre for Advancement in Inclusive and Special Education, The University of Hong Kong, Hong Kong, China; ^4^Department of Early Childhood Education, The Education University of Hong Kong, Hong Kong, China; ^5^Child Health Evaluative Sciences, The Hospital for Sick Children, Toronto, ON, Canada

**Keywords:** poor reader, reading, phonological awareness, working memory, music, Chinese, preschool

## Abstract

**Introduction:**

This study compared the phonological, musical, and general cognitive profiles of early-emerging poor, average, and good readers.

**Methods:**

We assessed Cantonese preschool children on Chinese word reading, phonological awareness, lexical tone awareness, musical rhythm perception, musical pitch perception, working memory, and non-verbal intelligence.

**Results:**

Early-emerging poor readers exhibited poorer phonological awareness than early-emerging average and good readers, whereas the latter two groups did not differ significantly. In the working memory task, early-emerging good readers outperformed both early-emerging average and poor readers, who performed similarly. No significant group differences were found in lexical tone awareness, musical rhythm perception, musical pitch perception, or non-verbal intelligence.

**Discussion:**

The results reflect phonological deficits in early-emerging poor readers. Furthermore, phonological awareness and working memory were useful for identifying early-emerging poor and good readers, respectively. Clinically, these findings imply that early-emerging poor readers may benefit most from initial phonological awareness training, followed by working memory training. Moreover, working memory training may also be beneficial for early-emerging average readers seeking to improve their Chinese word reading.

## 1 Introduction

Globally, 9 to 17% of school-age children are classified as poor readers (Catts et al., 2012; Gao et al., 2019). Despite having normal intelligence, these children exhibit word reading difficulties. Decades of research have assessed their phonological, musical, and general cognitive profiles, facilitating early identification and intervention (e.g., Chan and Siegel, 2001; Chiang et al., 2020; Dawes et al., 2015; McBride-Chang et al., 2012; Wang and Chen, 2025). However, despite progress, a group of children is often overlooked in this process—those whose word reading difficulties emerge early at preschool. Early-emerging poor readers pose a significant challenge for early identification and intervention because current findings are based on the school-age population. From a theoretical perspective, studying these children can extend existing evidence to the preschool age. To achieve this, we compared the phonological, musical, and general cognitive profiles of early-emerging poor, average, and good readers of Chinese.

### 1.1 Poor readers and early-emerging poor readers

There are various ways to conceptually define poor readers, reflecting differences in focus and criteria across research contexts. In the context of reading comprehension, the Simple View of Reading posits that word reading and language comprehension are two fundamental skills (Hoover and Gough, 1990). Accordingly, reading comprehension difficulties can stem from difficulties in word reading (*dyslexic readers*), language comprehension (*poor comprehenders*), or both (*poor readers*). Studies adopting this framework often define poor readers as children exhibiting word reading and language comprehension difficulties (Cain and Oakhill, 2006; Georgiou et al., 2008; Kelso et al., 2022), though *generally poor readers* is also used (Eckert et al., 2018; Kelso et al., 2007). Some research, however, refers to poor readers simply as children with reading comprehension difficulties, regardless of the underlying causes (Deng et al., 2019).

In contrast, the present study focuses on word reading rather than reading comprehension. Within this area, poor readers are often conceptually defined as children who struggle to read aloud written words despite having normal intelligence (Chan and Siegel, 2001; Cheema et al., 2023; McBride-Chang et al., 2012; Tong et al., 2015). Although specific criteria may vary across studies, many operationally define poor readers as those scoring in the lowest 25th percentile on a word reading task within their sample (McBride-Chang et al., 2012; Liu et al., 2010; Tong et al., 2015).

While the term *developmental dyslexia* may be considered for our study, we prefer to use *poor readers* for several reasons. First, developmental dyslexia is an official medical diagnosis that requires assessment by healthcare professionals, typically at age 7 or 8 (Child Assessment Service, 2018). Using this term would imply that participants are at least school-aged, which poses challenges for understanding early-emerging word reading difficulties during preschool years. Additionally, diagnostic criteria for dyslexia vary across classification systems (e.g., ICD-11, DSM-5) and cultural contexts, complicating cross-study comparisons (Folco et al., 2021). In contrast, the concept of poor reader is more straightforward to operationalize and is more appropriate for preschool populations.

Throughout the decades, much effort has been devoted to identifying the potential deficits underlying word reading difficulties at school age. As reviewed later, existing studies have largely compared poor readers and normally achieving readers across various constructs related to word reading. Across different language populations, poor readers have consistently been found to underperform compared to normally achieving readers in phonological awareness (Carrol and Breadmore, 2017; Cheema et al., 2023; McBride-Chang et al., 2012; Wang and Chen, 2025). More recently, poor readers have also been shown to have poorer musical rhythmic and pitch perception than normally achieving readers (Chiang et al., 2020; Wang and Chen, 2025). Additionally, they may exhibit deficits in working memory (Chan and Siegel, 2001). Although most of these studies are cross-sectional with limited causal inference, they have identified a number of phonological, musical, and general cognitive skills that may underlie word reading difficulties in poor readers. These findings have significant practical implications, contributing to early screening and intervention efforts for word reading difficulties (Winn et al., 2020).

Despite effort, a group of children has been overlooked in the literature. Technically, poor readers can only be identified after they have begun learning to read. In most countries, reading instruction starts at elementary school, so existing research on poor readers is predominantly based on school-age children (Catts et al., 2012; Parrila et al., 2005; Wang and Chen, 2025). In contrast, children in Hong Kong typically begin learning to read at preschool (Chow, 2018; Fung and Chung, 2019). From the second year of preschool until the third, children are systematically taught to read and write Chinese words. By the end of their third year, most preschool children can read approximately 150 to 200 Chinese words (Chung and McBride-Chang, 2011). Conceivably, as in the school-age population, there should be some preschool children in Hong Kong who struggle with word reading. Noting that the term *early poor readers* has already been used by Catts et al. (2012) to describe first-grade poor readers, we refer to this group of preschool children as *early-emerging poor readers*. If left untreated, early-emerging poor readers may enter elementary school with their existing word reading difficulties and later be identified as poor readers at school age (McBride-Chang et al., 2012).

From theoretical and practical perspectives, early-emerging poor readers create a unique context for investigating the early emergence of word reading difficulties and understanding the phonological, musical, and cognitive profiles from a much earlier age. Given that our early-emerging poor readers are from Hong Kong, our review primarily focuses on empirical studies related to poor readers of Chinese, complemented by theoretical frameworks that are in principle applicable across languages (Goswami, 2011; Patel, 2008; Perfetti and Hart, 2002). Hereafter, the terms *early-emerging poor readers* and *poor readers* specifically refer to those of Chinese, unless otherwise specified.

### 1.2 Phonological profile of early-emerging poor readers

Our first hypothesis is that early-emerging poor readers have poor phonological awareness. Phonological awareness refers to the ability to analyze and manipulate segmental phonological units of words, such as syllables, onset-rimes, and phonemes (Gillon, 2004). It is the foundation for reading across languages, and this also applies to non-alphabetical languages, such as Chinese (McBride-Chang et al., 2012; Perfetti et al., 1992). In Hong Kong, preschool reading instruction mainly adopts a whole-word approach (Chan and Siegel, 2001; Chow, 2018; Chung and McBride-Chang, 2011). For example, preschool teachers teach the Chinese word “貓” (*cat*) by showing its orthographic form together with its phonological form (/mau1/) and a description of its meaning (*an animal that meows and likes eating fish*). Unlike other Chinese-speaking regions, teachers in Hong Kong do not utilize any phonetic alphabetical system (e.g., *Hanyu* P*inyin* in mainland China and Z*huyin Fuhao* in Taiwan) to represent the pronunciation of Chinese words. Thus, children in Hong Kong rely on rote memorization and the mapping of phonological (e.g., /mau1/) and orthographic forms (e.g., 貓) of the words.

Although children in Hong Kong do not rely on phonetic alphabets to read Chinese, they still need to establish phonological representations of words (Chow, 2018). For example, if they have a poor phonological representation of the word (貓), they will experience difficulties reading it aloud (/mau1/). According to the lexical quality hypothesis, poor phonological awareness can lead to impoverished phonological representations, hindering children’s ability to read Chinese words aloud accurately (Perfetti, 2007; Perfetti and Hart, 2002). Moreover, forming phonological representations is more cognitively demanding for children with poor phonological awareness. As such, fewer cognitive resources are left to support other cognitively demanding processes such as visually analyzing complex orthographs (e.g., 貓). Indeed, phonological awareness has been found to predict Chinese word reading in preschool children in Hong Kong (Chow et al., 2005; McBride-Chang et al., 2004).

Previous studies on poor readers have provided some support for our hypothesis (McBride-Chang et al., 2012; Tong et al., 2015). Conducted in Hong Kong, a longitudinal study tracked Chinese children from K3 to Grade 4 (McBride-Chang et al., 2012). At Grade 4, poor readers were identified based on their composite Chinese word reading scores in Grades 3 and 4. Overall, these poor readers underperformed compared to normally achieving readers on phonological awareness. In a later study, poor readers also exhibited lower phonological awareness scores than normally achieving readers, although the difference was not statistically significant, possibly due to their small sample size (*n* = 25) (Tong et al., 2015). More recently, a study conducted in Taiwan found that poor readers underperformed on phonological awareness compared to normally achieving readers (Wang and Chen, 2025). It is important to note that different teaching methods are employed in Hong Kong (whole-word approach) and Taiwan (Zhuyin Fuhao), so the findings from Taiwan may not directly apply to Hong Kong. However, since the script being learned is the same, these results can still provide at least partial support for our study.

In addition to phonological awareness, lexical tone awareness is also an important skill in Chinese word reading. Lexical tone awareness refers to the ability to analyze and distinguish the pitch patterns of syllables (i.e., lexical tone) (Burnham et al., 2011; Tong et al., 2015). For example, the Cantonese syllable /si/ can represent at least six different meanings depending on its lexical tone, e.g., 絲 /si1/ *silk*, 史 /si2/ *history*, 試 /si3/ *try*, 時 /si4/ *time*, 市 /si5/ *market*, and 是 /si6/ *yes*. According to Cantonese corpus analysis, lexical tone has a high functional load in signaling lexical contrasts (Oh et al., 2015). Regarding preschool children in Hong Kong, lexical tone awareness has been found to predict Chinese word reading even after controlling for age, non-verbal intelligence, phonological awareness, speeded naming, and receptive vocabulary (McBride-Chang et al., 2008; Yeung and Ganotice, 2014).

Although no research has tested early-emerging poor readers, studies with school-age children suggest that poor readers tend to have deficits in lexical tone awareness (Chan and Siegel, 2001; Tong et al., 2015; Wang and Chen, 2025). In an early study, poor readers were less able to discriminate lexical tones compared to normally achieving readers (Chan and Siegel, 2001). More recently, a study found that poor readers performed worse than normally achieving readers in lexical tone awareness, although the difference was not statistically significant (Tong et al., 2015). In a Taiwan-based study, poor readers significantly underperformed compared to normally achieving readers in lexical tone awareness (Wang and Chen, 2025).

### 1.3 Musical profile of early-emerging poor readers

Our second hypothesis is that early-emerging poor readers exhibit poor musical rhythm and pitch perception. The temporal sampling framework posits that word reading difficulties can be attributed to impaired rhythmic processing, which may be reflected in poor musical rhythm perception (Goswami, 2011). Musical rhythm refers to the arrangement of sounds and silences of different durations that creates a regular and repeated pattern of beats or pulses over time (Hayes, 1989). In speech rhythm, the rise time and amplitude envelope provide essential acoustic information that helps listeners segment continuous acoustic signals into syllables, a process essential for developing phonological awareness (Goswami, 2011). According to this framework, children with impaired rhythmic processing are less efficient at syllabic segmentation, which can hinder the development of phonological awareness and, consequently, word reading (Goswami, 2011).

Recent evidence suggests that the temporal sampling framework may apply to poor readers of Chinese (Chiang et al., 2020; Wang and Chen, 2025). Compared with normally achieving readers, poor readers of Chinese showed a diminished magnetoencephalographic (MEG) response to rhythmical violations (Chiang et al., 2020). In a more recent behavioral study, poor readers of Chinese were less accurate than normally achieving readers in discriminating musical rhythm (Wang and Chen, 2025).

In addition to musical rhythm, poor readers have also been found to exhibit deficits in musical pitch perception. Acoustically, musical pitch and lexical tones are both pitch variations (Choi, 2020; Choi et al., 2017a, 2017b, 2018; Yiu, 2013). The resource-sharing framework posits that the processing of musical pitch and lexical tone shares at least partially overlapping neural resources (Patel, 2012). This framework suggests that poor lexical tone perception observed in poor readers may be linked to poor pitch sensitivity, as reflected in poor musical pitch perception (Chan and Siegel, 2001). Supporting our hypothesis, a Taiwan-based study found that poor readers were less accurate than normally achieving readers in the simultaneous perception of musical pitches (Wang and Chen, 2025).

### 1.4 General cognitive profile of early-emerging poor readers

Our third hypothesis is that early-emerging poor readers have poor working memory and non-verbal intelligence. As explained, preschools in Hong Kong adopt a whole-word approach for Chinese reading instruction (Chan and Siegel, 2001; Chow, 2018). Thus, children need to memorize phonological, orthographic, and semantic information about the word (Chung and McBride-Chang, 2011). After establishing stable phonological, orthographic, and semantic representations, children must also form interconnections between them (Perfetti and Hart, 2002). These processes are cognitively demanding, especially when children need to rely on rote memorization. Our hypothesis is exemplified by a previous study that found a significant correlation between working memory and Chinese word reading in preschool children in Hong Kong (Chung et al., 2017).

Although research on early-emerging poor readers is lacking, a Hong Kong-based study found that poor readers performed worse than normally achieving readers on a short-term memory task, which is a component of working memory responsible for retaining information during mental operations (see Baddeley, 2003). However, this short-term memory task required children to remember Chinese orthographs (So and Siegel, 1997), raising the possibility that the observed deficits were influenced by poor Chinese word reading itself. To address this circularity issue, the current study employed a working memory task that did not involve reading (Choi et al., 2025; Bunting, 2006).

Besides working memory, general intelligence is a well-established predictor of learning success; therefore, we also included it in our cognitive measures (Lozano-Blasco et al., 2022; Mangels et al., 2006). In a previous study, English poor readers were found to have lower non-verbal intelligence than typically achieving readers (Ellis et al., 1996). However, unlike in many studies, their definition of poor readers did not exclude individuals with below-normal intelligence, meaning some of their poor readers fell outside the normal intelligence range. Although their results are difficult to interpret, it is plausible that, even within the normal range, preschool children with relatively lower intelligence may underperform compared to their higher-intelligence peers. To avoid confounding effects related to language, we assessed non-verbal intelligence rather than verbal intelligence (Raven, 2008).

### 1.5 Early-emerging average and good readers

Besides the scarcity of research on early-emerging poor readers, existing research has seldom differentiated between average and good readers. In previous studies, poor readers were compared with a group of normally achieving readers (e.g., Chan and Siegel, 2001; McBride-Chang et al., 2012; Tong et al., 2015). While poor readers were those who scored in the bottom 25% on a word reading task, normally achieving readers were often defined as those who scored within the top 75% (McBride-Chang et al., 2012), 70% (Chan and Siegel, 2001), or 50% (Tong et al., 2015). Consequently, the word reading performance range among normally achieving readers is quite broad. By further subdividing this group into average readers and good readers, we can gain a more nuanced understanding of the phonological, musical, and general cognitive profiles at different achievement levels. In practice, this differentiation may inform targeted interventions—helping poor readers catch up to become average readers, and supporting average readers in advancing to become good readers. Such insights are crucial for designing tailored educational strategies that promote reading development across different achievement levels.

In short, our study aimed to examine the phonological, musical, and general cognitive profiles of early-emerging poor readers, average readers, and good readers. Our hypotheses were that early-emerging poor readers would perform worse than early-emerging average and good readers across measures of phonological awareness, lexical tone awareness, musical rhythm perception, musical pitch perception, working memory, and non-verbal intelligence. Empirically, this study investigated:

1. Whether early-emerging poor, average, and good readers significantly differed in phonological and lexical tone awareness.
2. Whether early-emerging poor, average, and good readers significantly differed in musical pitch and rhythm perception.
3. Whether early-emerging poor, average, and good readers significantly differed in working memory and non-verbal intelligence.

## 2 Materials and methods

### 2.1 Participants

Initially, 104 Cantonese-speaking preschool (K2 to K3) children (*M_age_* = 4 years and 10 months; *SD* = 9 months) from preschools in Hong Kong were enrolled in our study. All children spoke Cantonese as their mother tongue, used it as their primary language, and had never lived outside Hong Kong. The parents confirmed that they had communicated with their children in spoken Cantonese since birth. According to parental and school reports, none of the children had a current or past diagnosis of any neurodevelopmental disorders (e.g., autism spectrum disorder), intellectual disabilities, or deafness. Parental written consent was obtained before testing the children. Upon completion of the study, the children were awarded a certificate and a 50 HKD book coupon. This study was approved by the Human Research Ethics Committee of the University of Hong Kong. Prior to data collection, written informed consents and oral assents were obtained from the parents and children, respectively.

Next, we classified the participants into early-emerging poor, average, and good readers based on their performance in our Chinese word reading task. To ensure consistency and comparability with previous research, we adopted the same criteria used in prior studies to identify poor readers (e.g., Chan and Siegel, 2001; McBride-Chang et al., 2012; Tong et al., 2015). Specifically, poor readers were defined as children scoring in the lowest 25th percentile of all participants. Following recent studies, average readers were those who scored above the 50th percentile but below the 75th percentile for Chinese word reading. The 50th percentile cutoff has been shown to be sufficiently rigorous for excluding children with mild reading difficulties (Liu et al., 2010; Tong et al., 2015). Good readers were those who scored at or above the 75th percentile. The 75th percentile criterion was considered reasonably liberal given our sample size. Nineteen participants who scored between the 25th and 50th percentiles were excluded, as they did not meet any of the grouping criteria.

The final sample comprised 85 children (*M_age_* = 4 years and 9 months; *SD* = 9 months), with 32 early-emerging poor readers (21 boys and 11 girls), 20 average readers (10 boys and 10 girls), and 33 good readers (15 boys and 18 girls). An *a priori* power analysis indicated that a total sample size of 81 would provide 95% power to detect the main effect of group with the minimum effect size (d = 0.64) reported in previous research (Wang and Chen, 2025), at a 0.05 significance level. Therefore, we consider our study sufficiently powered.

To verify the group classifications, we conducted a one-way ANCOVA on Chinese word reading with group (early-emerging poor, average, and good readers) as the between-subjects factor and age as the covariate. The main effect of group was significant, *F*(2, 81) = 779.77, *p* < 0.001, η^2^ = 0.95. Then, we conducted pairwise comparisons with Bonferroni adjustments for the degrees of freedom. Consistent with our group classification, early-emerging poor readers had the lowest mean accuracy in Chinese word reading among the three groups, *ps* < 0.001. Moreover, early-emerging good readers had a significantly higher mean accuracy than early-emerging average readers, *p* < 0.001.

For socioeconomic status, we examined family income but parents had the option not to disclose this information. Based on the 63 responses received, we conducted a one-way ANOVA on family income, with group (early-emerging poor, average, and good readers) as the between-subjects factor. The main effect of group was not significant, *p* = 0.538, indicating that the three groups did not differ significantly in their family income.

### 2.2 Procedure

Each child was individually tested by a research assistant in a quiet room at a local preschool or university. The research assistant holds a bachelor’s degree in linguistics and has experience in working with children. All auditory stimuli were played at an appropriate volume through headphones designed for children. As detailed below, there were seven tasks. Based on a Latin square design, the tasks were presented in a counterbalanced order across seven lists, with each list assigned to 12–13 children. Breaks were provided between the tasks. The total testing time was approximately 75–90 min for each child, split across two or three sessions held no more than two weeks apart.

#### 2.2.1 Chinese word reading

We used an adapted version of the Chinese word reading task (McBride-Chang et al., 2004). The task comprised of a sheet of paper with 30 single-syllable words and 25 two-syllable words (see Table 1). To ensure that the task is developmentally appropriate for preschool children, the words were selected from extracurricular Hong Kong preschool books. The words were arranged in ascending order of word frequency. The children were instructed to read each word aloud. The experimenter assessed the accuracy of the pronunciation for each word without providing feedback to the child. A trial was deemed incorrect if the child indicated that they did not know how to pronounce the word or if any phoneme or tone was mispronounced or omitted. One point was awarded for each correct trial, whereas incorrect trials received no points. Percentage accuracy was determined by dividing the number of correct test trials by 55. Based on our pilot data from preschool children, the scores followed a normal distribution without floor/ceiling effects, indicating that the task effectively captures the variability in word reading within this age group. Testing was stopped if the child failed to read 10 consecutive items aloud. The task had high internal consistency (Cronbach’s alpha = 0.98).

**Table 1:** Chinese word reading list.

山	牛	花	車	水
爸	哥	色	風	早
生	狗	媽	手	秋
我	樹	球	藍	好
兒	飛	多	快	個
的	蕉	去	在	說
香港	市場	新年	星期	耳朵
中午	眼睛	晚上	老師	房屋
美麗	衣服	鼻子	再見	禮物
蜜蜂	書包	電腦	馬路	糖果
你們	家庭	飲食	牙刷	海灘

The wordlist went from left to right, and top to bottom.

#### 2.2.2 Phonological awareness

We used a revised version of the Chinese phonological awareness task (Choi et al., 2016, 2025). This task comprised of two subtests: syllable deletion and onset phoneme deletion. The syllable deletion subtest contained 20 trials in which children were instructed to verbalize a trisyllabic word or pseudoword by omitting the first, second, or third syllable (e.g., say /k^h^un1 mɛk1 p^h^au6/ without /mɛk1/). In the onset phoneme deletion subtest, there were 10 trials in which the children were tasked with reproducing a monosyllabic word or pseudoword without the initial phoneme (e.g., say /sai3/ without /s/). Tone matching was not required in these subtests, because they focused solely on segmental information. Each subtest included two practice trials with feedback, and one point was awarded for each correctly completed test trial. Percentage accuracy was computed by dividing the number of correct test trials by 30. Testing was stopped if the child failed six consecutive items. The task had high internal consistency (Cronbach’s alpha = 0.96).

#### 2.2.3 Lexical tone awareness

We adopted the odd-one-out task from a previous study (Cheng, 2018). In this task, children were initially presented with three monosyllabic words (e.g., /kai1/ /pai2/ /ts^h^ai2/) in each trial. Subsequently, they were required to identify the word that had a different tone from the others (e.g., / kai1/). The task was divided into two blocks. In one block, the rime remained consistent within each trial (e.g., /kai1/ /pai2/ /ts^h^ai2/), while in the other block, the rime varied within each trial (e.g., /kɐu2/ /hai4/ /jy2/). The task commenced with four practice trials that offered feedback, followed by 36 test trials without feedback. Each correctly answered test trial earned one point. Percentage accuracy was computed by dividing the number of correct test trials by 36. There was no discontinuation rule in this task. The task had satisfactory internal consistency (Cronbach’s alpha = 0.71).

#### 2.2.4 Musical rhythm perception

We adopted the abbreviated rhythm test from the Montreal Battery of Evaluation of Musical Abilities (*MBEMA*, Peretz et al., 2013). In each trial, children were presented with two rhythmic phrases separated by a 1500ms inter-stimulus interval (ISI). Subsequently, they were required to determine whether the two phrases were identical or different. In all *different* trials, the second phrase contained altered durations of two adjacent tones, resulting in a change in rhythm while maintaining the number of notes and original meter. The task included three practice trials with feedback and 20 test trials without feedback. Each correct test trial earned one point. Percentage accuracy was calculated by dividing the number of correct test trials by 20. There was no discontinuation rule in this task. The MBEMA had satisfactory internal consistency (Cronbach’s alpha = 0.62).

#### 2.2.5 Musical pitch perception

We adopted the abbreviated melody test from MBEMA (Peretz et al., 2013). In each trial, children were presented with two melodic phrases separated by a 1500ms ISI. Subsequently, they were tasked with determining whether the two phrases were the same. On each *different* trial, the second melodic phrase retained the original key of the first phrase but included either an off-key note, a changed note altering the pitch direction of surrounding intervals, or a changed note affecting intervals. The task contained three practice trials with feedback and 20 test trials without feedback. Each correct test trial was awarded one point. Percentage accuracy was calculated by dividing the number of correct test trials by 20. There was no discontinuation rule in this task. The MBEMA had satisfactory internal consistency (Cronbach’s alpha = 0.62).

#### 2.2.6 Working memory

We utilized an adapted version of the running memory span task (Choi et al., 2025) that was originally designed by Bunting (2006). In each trial, the children were presented with a sequence of digital triplets, such as 216, 858, and 712. They were then required to recall the final digit of each triplet; for instance, 682. The number of sets was gradually increased from two to eight. The task included two practice trials with feedback followed by 21 test trials without feedback. Testing was discontinued if the child provided three consecutive incorrect responses. Percentage accuracy was determined by dividing the number of correct test trials by 21. Testing was stopped if the child failed three consecutive items. The task had high internal consistency (Cronbach’s alpha = 0.82).

#### 2.2.7 Non-verbal intelligence

We adopted an abbreviated version of Raven’s Coloured Progressive Matrices (Raven, 2008). Each trial contained an unfinished visual pattern accompanied by several choices for completion. The children were asked to choose the option that most appropriately finished the visual pattern. The assessment was comprised of 12 trials. The total number of accurate test trials was tallied and divided by 12 to determine percentage accuracy. There was no discontinuation rule in this task. The task had satisfactory internal consistency (Cronbach’s alpha = 0.77).

## 3 Results

**Figure 1:**
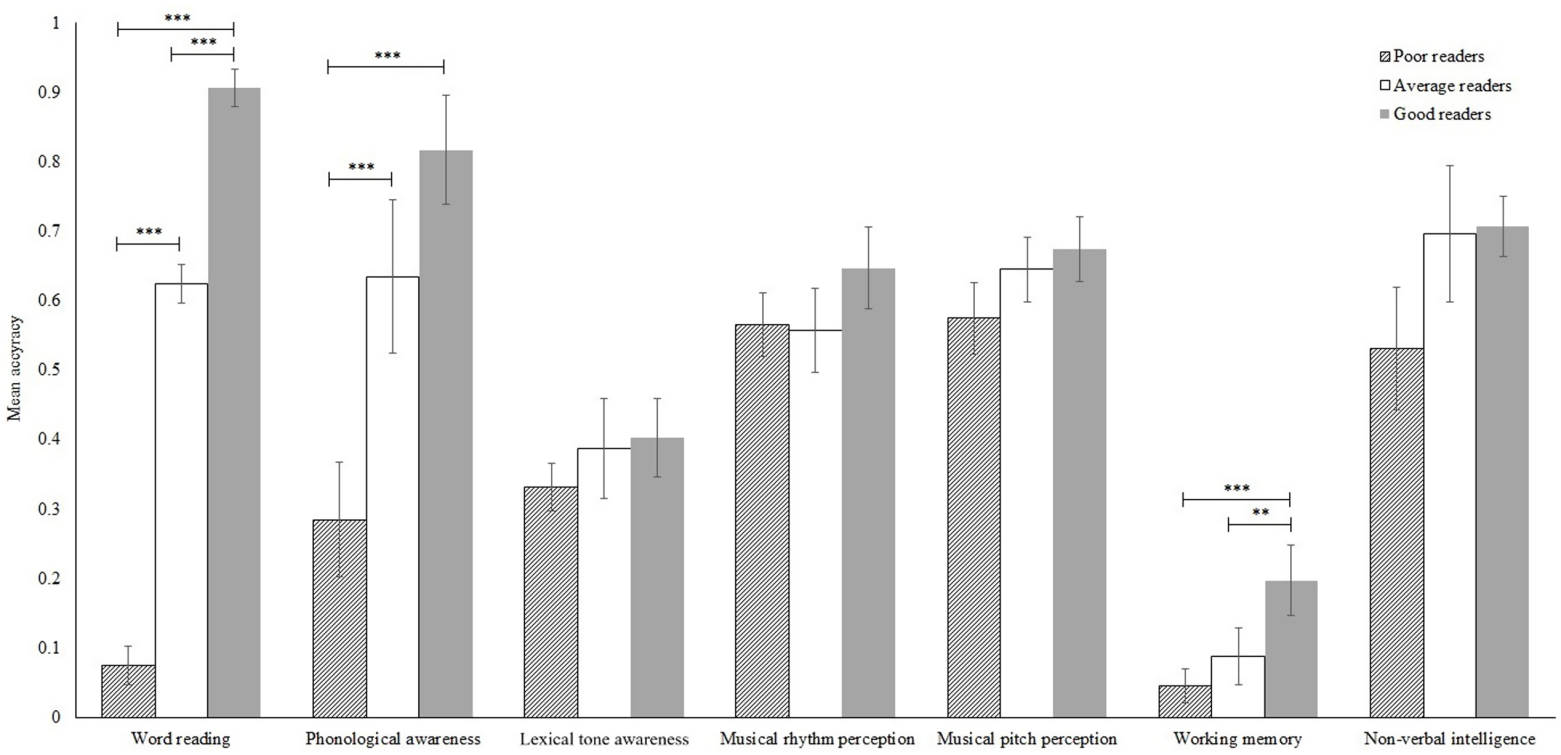
Mean accuracies of the early-emerging poor, average, and good readers in all the tasks. The error bars represent 95% confidence intervals.

Figure 1 summarizes the mean accuracies of the early-emerging poor, average, and good readers for all the tasks. Prior to the main analyses, we conducted one-sample *t*-tests to evaluate whether participants performed significantly above floor or chance in all tasks. For word reading, phonological awareness, and working memory, participants performed significantly above zero, *ps* < 0.001. For the tasks that involved choices, i.e., lexical tone awareness, musical rhythm perception, musical pitch perception, and non-verbal intelligence, the participants performed significantly above their respective chance levels, *ps* < 0.003.

### 3.1 Phonological and lexical tone awareness

Our first hypothesis was that early-emerging poor readers exhibit poorer phonological and lexical tone awareness compared to early-emerging average and good readers. To test this hypothesis, we conducted a one-way MANCOVA on phonological and lexical tone awareness with group (early-emerging poor, average, and good readers) as the between-subjects factor and age as the covariate. The main effect of group was significant, Ʌ_Wilks’_ = 0.63, *F*(4, 160) = 10.23, *p* < 0.001, η_p_^2^ = 0.20. Specifically, univariate *F* tests showed a significant main effect of group on phonological awareness, *F*(2, 81) = 23.20, *p* < 0.001, η_p_^2^ = 0.36, but not on lexical tone awareness, *p* = 0.459. For the main effect of group on phonological awareness, we further conducted pairwise comparisons with Bonferroni adjustments. Relative to early-emerging average and good readers, early-emerging poor readers had significantly poorer phonological awareness, *ps* < 0.001. However, early-emerging average and good readers performed similarly, *p* = 0.150.

### 3.2 Musical rhythm and pitch perception

Our second hypothesis was that early-emerging poor readers exhibit poorer musical rhythm and pitch perception compared to early-emerging average and good readers. We conducted a one-way MANCOVA on musical rhythm and pitch perception with group (early-emerging poor, average, and good readers) as the between-subjects factor and age as the covariate. The analysis showed that the main effect of group was not significant, *p* = 0.388. Statistically, traditional MANCOVA can only reject or fail to reject a null hypothesis (Ly et al., 2019). In the above analysis, the lack of a significant main effect of group does not necessarily mean that it was absent.

To tackle this statistical issue, we conducted supplementary Bayesian analyses with JASP 0.17.3 (JASP Team, 2023). Unlike null hypothesis testing, Bayesian hypothesis testing quantifies evidence for both alternative and null hypotheses (Dienes, 2016). Thus, this could provide evidence for the absence of the main effect of group.

First, we conducted a Bayesian ANCOVA on musical rhythm perception, with group (early-emerging poor, average, and good readers) as the fixed factor and age as the covariate. We used a uniform prior that is relatively objective (Jaynes, 2003). In total, there were four models (see Table 2). The best fit model was the model with age only. Based on the Bayes factors (BF_01_), the best fit model was 2.76–9.64 times more likely than the other models.

**Table 2:** Model comparison relative to the best-fit model of musical rhythm perception.

Models	P(M)	P(M|data)	BF_M_	BF_01_	Error %
Age (best-fit)	0.250	0.613	4.749	1.000	
Group + Age	0.250	0.222	0.857	2.758	1.068
Group	0.250	0.101	0.338	6.048	0.026
Null	0.250	0.064	0.204	9.638	4.32 × 10^−4^

Second, we conducted the same Bayesian ANCOVA on musical pitch perception. Again, the best fit model was the model with age only (see Table 3). Based on BF_01_, the best fit model was 5.63–23932.47 times more likely than the other models. Collectively, none of the best fit models contained group as the fixed factor, supporting the absence of group differences in musical rhythm and pitch perception.

**Table 3:** Model comparison relative to the best-fit model of musical pitch perception.

Models	P(M)	P(M|data)	BF_M_	BF_01_	Error %
Age (best-fit)	0.250	0.849	16.854	1.000	
Group + Age	0.250	0.151	0.533	5.626	0.957
Group	0.250	1.92 × 10^−4^	5.75 × 10^−4^	4432.87	0.015
Null	0.250	3.55 × 10^−5^	1.06 × 10^−4^	23932.47	0.005

### 3.3 Working memory and non-verbal intelligence

Our third hypothesis was that early-emerging poor readers have poorer working memory and non-verbal intelligence than early-emerging average and good readers. We conducted a one-way MANCOVA on working memory and non-verbal intelligence with group (early-emerging poor, average, and good readers) as the between-subjects factor and age as the covariate. The main effect of group was significant, Ʌ_Wilks’_ = 0.83, *F*(4, 160) = 3.97, *p* = 0.004, η_p_^2^ = 0.09. Specifically, univariate *F* tests showed a significant main effect of group on working memory, *F*(2, 81) = 6.32, *p* = 0.003, η_p_^2^ = 0.14, but not on non-verbal intelligence, *p* = 0.123. For the main effect of group on working memory, we further conducted pairwise comparisons with Bonferroni adjustments. Early-emerging good readers had significantly better working memory than early-emerging average and poor readers, *ps* = 0.019 and 0.005. However, the latter two groups did not differ significantly, *p* = 1.00.

To supplement our findings, we re-conducted the main analyses with a more stringent criterion for early-emerging poor and good readers (i.e., the lowest 10th and the highest 10th percentiles), and the results were consistent with the main analyses.

## 4 Discussion

The present study compared the phonological, musical, and general cognitive profiles of early-emerging poor, average, and good readers. Compared with early-emerging average and good readers, early-emerging poor readers had poorer phonological awareness. While early-emerging good readers outperformed early-emerging average and poor readers in the working memory task, the latter two groups did not differ significantly. In addition, there were no group differences in lexical tone awareness, musical rhythm perception, musical pitch perception, and non-verbal intelligence.

### 4.1 Phonological profile

Our first finding is that early-emerging poor readers exhibited poorer phonological awareness compared to early-emerging average and good readers. This aligns with the prominent view that poor readers across different language populations exhibit a phonological deficit (Carrol and Breadmore, 2017; Cheema et al., 2023; McBride-Chang et al., 2012). By testing early-emerging poor readers, our study extends the developmental timeline of this phonological deficit from school age to preschool age. Practically, our findings imply that phonological awareness could be used to screen and intervene with preschool children with early word reading difficulties, at least in Chinese.

Furthermore, our findings indicate that poor phonological awareness can be a hallmark of word reading difficulty even in non-alphabetical languages. This aligns with previous research on poor readers in Taiwan (Wang and Chen, 2025). Unlike Taiwan, where Zhuyin Fuhao is part of reading instruction, Hong Kong adopts a whole-word approach without using alphabetic systems (Chan and Siegel, 2001; Chow, 2018; Chung and McBride-Chang, 2011). In addition to extending the timeline to preschool, our study demonstrates that even when learning to read a non-alphabetical language in a non-alphabetical way, poor phonological awareness remains a key indicator of word reading difficulties.

Why is this the case? With poor phonological awareness, early-emerging poor readers are less able to form stable phonological representations, resulting in difficulties reading Chinese words aloud (Perfetti, 2007; Perfetti and Hart, 2002). While children with normal phonological awareness experience more automated phonological processing, those with poor phonological awareness may require extra cognitive resources for phonological processing. Consequently, fewer cognitive resources are available to support other mentally demanding processes, such as orthographic analysis and forming connections between phonological, orthographic, and semantic representations (Li et al., 2012).

Interestingly, our findings slightly diverge from a longitudinal study conducted in Hong Kong (McBride-Chang et al., 2012). While their overall analysis found that poor readers exhibited poorer phonological awareness than normally achieving readers, their nuanced analysis revealed that the two groups had similar levels of phonological awareness during preschool age. This appears to contradict our finding that preschool-aged early-emerging poor readers exhibit poor phonological awareness. This discrepancy may stem from methodological differences. In the previous study, poor readers were identified at Grade 4 (McBride-Chang et al., 2012), and these children might not have been early-emerging poor readers during their preschool years. Notably, research on alphabetic languages has reported a significant prevalence of late-emerging poor readers, whose word reading difficulties only manifest in late elementary school (see Catts et al., 2012). In contrast, our study identified early-emerging poor readers based on their preschool word reading performance, and those with word reading difficulties at preschool were concurrently found to have poor phonological awareness.

Extending previous studies, we further differentiated normally achieving readers as early-emerging average readers and good readers (McBride-Chang et al., 2012; Tong et al., 2015). In our study, phonological awareness only differentiated early-emerging poor readers from both early-emerging average and good readers. In other words, early-emerging average and good readers exhibited similar levels of phonological awareness. This suggests that phonological awareness may be a limiting factor primarily when Chinese word reading is poor. For children who have already achieved average word reading, improving phonological awareness might not significantly enhance their word reading. These findings imply that, at least in Chinese, phonological awareness training could be beneficial for helping early-emerging poor readers reach an average reading level. However, once children have surpassed this bottleneck and become early-emerging average readers, phonological awareness training may no longer be effective in helping them become early-emerging good readers.

In contrast to our hypothesis, early-emerging poor readers did not exhibit poor lexical tone awareness. This finding aligns with a study conducted in Hong Kong (Tong et al., 2015) but contrasts with earlier research (Chan and Siegel, 2001) and a recent study in Taiwan (Wang and Chen, 2025). Since the earlier study was conducted 23 years ago, it is possible that the phonological profile of poor readers in Hong Kong has changed over the decades (Chan and Siegel, 2001). A more recent study on school-age children in Hong Kong found no differences in lexical tone awareness between poor and normally achieving readers (Tong et al., 2015), indicating that our results are unlikely due to testing younger participants. Extending Tong et al. (2015), who did not differentiate between true and false negatives, our Bayesian analysis provided moderate evidence that early-emerging poor readers have normal lexical tone awareness (Lee and Wagenmakers, 2013). Regarding the study conducted in Taiwan, they tested older children (8 to 12 years old) who speak Mandarin, which has a different lexical tone system compared to Cantonese (Wang and Chen, 2025). Therefore, direct comparisons between their findings and ours are not possible.

### 4.2 Musical profile

Our second finding is that early-emerging poor readers did not exhibit poor musical rhythm and pitch perception. In a related study involving preschool children in Hong Kong, musical rhythm perception did not predict phonological awareness after controlling for age and general cognitive skills (Choi et al., 2025). Similarly, musical pitch perception did not predict lexical tone awareness. From a theoretical perspective, the past and present results directly contrast with the temporal sampling and resource-sharing frameworks, which predicted that deficits in musical rhythm and pitch processing would be evident in early-emerging poor readers (Goswami, 2011; Patel, 2012). However, data from second and third graders in Hong Kong suggest that auditory pitch discrimination predicts phonological and lexical tone awareness, which in turn predicts word reading (Zhang and McBride-Chang, 2014). Therefore, the temporal sampling and resource-sharing frameworks may only apply to Chinese children in Hong Kong after they have reached second or third grade. Future longitudinal research is needed to explore this possibility. As it stands, our results imply that musical rhythm and pitch perception may not be effective tools for screening early-emerging poor readers. Additionally, musical pitch and rhythm training may not benefit early-emerging poor readers, although such training might still be effective with school-age children (Bigand and Tillmann, 2022; Neves et al., 2022).

### 4.3 General cognitive profile

Unexpectedly, early-emerging poor readers performed comparably to early-emerging average readers on the working memory task. However, working memory can help us identify early-emerging good readers. In Hong Kong, preschool children are typically expected to learn at least 150 to 200 Chinese words (Chung and McBride-Chang, 2011). Unlike alphabetic systems, Chinese script requires children to memorize each orthograph individually and establish links with sounds and meanings (Perfetti, 2007; Perfetti and Hart, 2002). Compared to learning 26 alphabet letters in English, memorizing 150 to 200 orthographs is cognitively demanding. This process is further complicated by the visual complexity of some orthographs (e.g., 灘 /than1/ beach) and the presence of highly confusable orthographs (e.g., 因–困, /jɐn1/−/k^w^ɐn3/, *because*–*trapped*), which children must differentiate and remember. Children with high working memory have more cognitive resources to encode and map phonological, orthographic, and semantic information efficiently, thereby improving lexical quality and facilitating word reading (Perfetti and Hart, 2002).

Unlike the bottleneck case of phonological awareness, working memory seems to be a limiting factor of Chinese word reading at the higher end. While phonological awareness training may help early-emerging poor readers become early-emerging average readers, working memory training can help early-emerging poor and average readers achieve good reading. From a practical perspective, our findings indicate that phonological awareness training can be initially utilized for early-emerging poor readers, but as they progress, working memory training will be more helpful. Moreover, working memory training can also be applied to early-emerging average readers seeking to achieve better Chinese word reading. A future intervention study employing a carefully designed randomized controlled trial would provide causal evidence to substantiate our claim.

### 4.4 Limitations and future directions

Our study has several limitations and avenues for future research. In Hong Kong, developmental dyslexia is typically diagnosed around the age of 7 to 8 (Child Assessment Service, 2018), and our participants were far too young for this diagnosis (*M*_age_ = 4 years 10 months). To situate into the dyslexia literature, future studies can test older children who have been professionally diagnosed with developmental dyslexia. To avoid prolong testing time, we did not assess other language-related skills such as vocabulary knowledge and morphological awareness (Liu et al., 2010). If time permits, future studies should assess a larger set of language-related skills. Lastly, our study is cross-sectional with limited causal inference. Longitudinal studies will enable us to clarify the causal nature of the relations between phonological awareness and word reading.

## 5 Conclusion

The present study extends the existing school-age literature by demonstrating that early-emerging poor readers exhibit weaker phonological awareness compared to early-emerging average and good readers. Additionally, we found that phonological awareness can distinguish early-emerging poor readers from early-emerging average and good readers, but it is not sufficient to identify early-emerging good readers. Conversely, working memory appears to be a useful tool for identifying early-emerging good readers. Practically, these findings imply that interventions for early-emerging poor readers may prioritize phonological training initially, followed by working memory training to further support word reading development. Moreover, working memory training could be beneficial for early-emerging average readers aiming to advance to early-emerging good readers.

## Data Availability

The datasets presented in this study can be found in online repositories. The names of the repository/repositories and accession number(s) can be found below: https://osf.io/879tv/?view_only=0d29a7c46d5f4f6ea8b5677dcb5bb37e (open science framework).

## Appendix

**Table A1:** Model comparison relative to the best-fit model of lexical tone awareness.

Models	P(M)	P(M|data)	BF_M_	BF_01_	Error %
Age	0.250	0.572	4.004	1.000	
Group + Age	0.250	0.198	0.739	2.891	0.002
Group	0.250	0.140	0.490	4.075	0.039
Null	0.250	0.090	0.298	6.333	2.127
